# Biometric Technology at the Borders of Citizenship: Identifying Technical Standards for Introducer-Based Remote Onboarding in Global Contexts of Statelessness, Nomadism, Displacement, and Refuge

**DOI:** 10.1515/ijdlg-2024-0010

**Published:** 2024-07-08

**Authors:** Riccardo Vecellio Segate

**Affiliations:** Trustworthy Digital Infrastructure for Identity Systems, The Alan Turing Institute, London, UK; Faculty of Economics and Business 3647 University of Groningen, Groningen, The Netherlands

**Keywords:** technical standards in biometrics, borderline citizenship, identity introducer, migrations, nomadism, displacement, statelessness, and refuge, remote identity onboarding

## Abstract

All throughout the so-called “Global South”, hundreds of millions of individuals from entire communities in the rural, poorer, or most peripheral areas are not officially recorded by the States they are citizens of or they habitually reside in. This is why several of such States are resorting to extensive and purportedly “universal” digital remote onboarding programs, pioneered by India’s Aadhaar, whereby individuals are centrally recorded onto a public database with their identity (and possibly citizenship) confirmed. Whenever paper documents are obsolete, inaccurate, deteriorated, or inexistent, individuals may have their identity confirmed through an “introducer”, who mediates between marginalised communities and central authorities and is entrusted by both with this delicate task. Introducers, however, cannot by themselves grant someone the status as “citizen”: they may at best confirm his or her existence and identity. These onboarding programs are enabled by wide-covering sets of technical standards, ranging from data protection and cybersecurity to interoperability, safety, disaster recovery, and business continuity. Meanwhile, similar technologies, relying on analogous standards, and fundamentally aimed at a similar purpose (that is, registering all those who fall within the prescriptive jurisdiction of a State), are deployed by border officials in the context of migration management – especially in “developed” countries. The “unofficial” and “outside-the-scope-of-the-law” components of said migratory patterns are growing exponentially due to combined effects of climate, insecurity, and geopolitical factors, increasingly originating “borderline” situations whereby identity and citizenship are challenged and contested: statelessness, refuge, nomadism (both traditional and “digital”), and internal displacement. Strikingly enough, discussions around what technical standards to adopt, and who should select them, as well as on what the role of “introducers” could be, towards the digital onboarding of individuals experiencing “borderline” configurations of citizenship are entirely neglected in socio-legal and security scholarship alike. Complemented with concrete policy proposals, the present work accepts the ambition to start bridging this gap.

## 1 Introduction

Unlawful, quasi-lawful, unregulated, unofficial, unrecorded, or in any way “borderline” migratory patterns seem to stage the perfect case-study for remote identity onboarding applications today. The “remote” component is essential when people are on the move, all the more so if they move through unofficial and perhaps even unlawful channels: when an asylum seeker flees from persecution, they1One could use “he” for reasons of practicality: it would accurately reflect the on-the-ground reality, with the overwhelming majority of cross-border migrants being (young) men. They tend to send remittances back to their families, and reunite (mostly lawfully) at a later stage in the destination country. might need to keep hiding from the pushing2In international migration law and especially international refugee law, the “pushing country” is a migrant’s country of origin, while the “pulling country” is their destination country. country’s state authorities; and when escaping “natural” disasters or climate threats, including upcoming conflicts for resources, they might be moving swiftly to escape those (Vecellio Segate 2022a). What is more, individuals in these situations might dislike the idea of having their data collected for governmental or para-governmental purposes – at least in the short run, that is, until they have reached a destination they consider safe, or the political regime they escape from has stipulated credible safeguards for them and their families.

In fact, conceptually, the XXI century has come a long way in identity management, with the normalisation of securitised borders becoming arguably the most characteristic trait of public order today. Throughout thousands of years of civilisation, humans have always been able to potentially relocate around the planet at will, either because there were no formal political borders at all, or because borders were not enforceable, and even when they were enforced, they proved porous and changed quite frequently. One way or the other, until the advent of satellites, facial recognition, radars, algorithms, and other technologies, borders’ formal enforcement was fraught with blind spots, turnarounds, and exceptions. This is no longer the case: States tend to keep their borders virtually unaltered for decades – when not centuries; meanwhile, satellite surveillance, physical fences, urban control, air and water patrolling, remote sensing, shared identity repositories, and especially the globe-wide deployment of biometric tracking are redefining the landscape of who can move and where to. To be sure, the extensive deployment of biometric tracking at States’ borders has been with us for almost two centuries already (Breckenridge 2014), but with the caveats that it was not operable remotely and it was neither digital, nor algorithmic: it is automation and remote operability that makes biometrics nearly inescapable today. Blind spots are becoming increasingly rare, and those who embark onto exploiting these residual fault lines in search of a more dignifying future are often criminalised and incarcerated as biopolitical waste (Corcodel and Fragkou 2023; Lesutis and Kaika 2024). This bears evident repercussions on the meaning of citizenship and the multi-layered fragmentation of our rather fragile planet based on transnational wealth-sorted classes and new migratory élites (Vecellio Segate 2022c; Walsh 2014).

Because the administration and recording of individuals’ identities, under the comfortable rhetorical tenet of their “protection”, is central to the very existence of a population and thus of a State itself, public international law (PIL) – as the legal regime applicable between sovereign States – has long been concerned with the question of citizenship and the bureaucratic control thereof (Chakrabarty 2021; Soomro 2023). Within this extremely broad landscape, issues of “borderline citizenship” such as statelessness, nomadism, displacement, and refuge have gained momentum as a core concern for public international lawyers, due to both their transnational nature (in most cases at least) and their inevitable interfaces with the human-rights and human-security agendas. In particular, recent scholarship (e.g. Jain 2022) has brilliantly unpacked the State as a *proactive producer* of borderline citizenship as opposed to a discourse traditionally focused on the State as a “humanitarian” agent whose interest would rest with closing such gaps. Public international lawyers are all the more concerned as the current post-Westphalian configuration of international relations displays the properties of a regulatorily captured regime which is fraught with exclusionary exceptionalisms, carveouts, and grey areas, where transnational capital flows thrive along with their reference élites (Buzan and Little 1999; Grell-Brisk 2018), but specific classes of “faceless” individuals often remain trapped in-between domestic laws, to no one State’s avail (Benhabib 2020). It seems appropriate to clarify from the outset that references here will be to *citizenship* and not to *nationality*: the two terms are too often confused, but the former strictly refers to the objective legal status of being recognised as a “citizen” by a State, while the latter broadly describes a sense of self-assessed ethno-cultural affiliation, bond, kinship, and belonging to a national community (von Rütte 2022, 11–57). This is not to say that *nationality* is completely alienated from entitlements and responsibilities, especially from a PIL perspective (Edwards 2014, 11; Macklin 2015, 224–225); but when it comes to rights and duties as delivered under positive domestic law, the status that articulates them is correctly defined as *citizenship*.

The “Global North” (GN) stands apparently uninterested in reducing the scope of global “borderline citizenship” phenomena, or at least in doing so in such a genuine way that those who find themselves in those conditions could accept and volunteer for. Developed nations’ contribution to “segregation by bordering” is so deep and cruel that lawyers and human rights litigators have most recently resorted to international-criminal-law arguments and strategies, usually confined to the most atrocious failures of mankind (Kalpouzos 2020). Different is the stance of first-impacted “developing” countries, where providing unregistered individuals with an identity (and possibly a legal status such as citizenship), and doing so *digitally*, features right at the core of the political agenda, starting with the massive, pioneering (however contested) Aadhaar system implemented in India. These States, however, lack the policy instruments as well as the know-how to address transnational “producers” of borderline citizenship as they (would) do with their own domestic borderline phenomena: remote digital onboarding in the context of borderline migration raises a series of challenges that cannot be successfully tackled by any jurisdiction alone, and where inter-State cooperation is made more difficult by the “high-politics” nature of today’s discourse on global migrations governance. Furthermore, it is worth problematising the “developmental” impetus impressed by (often GN-designed) digital identity systems to disadvantaged societies, where the hiatus between surveillance and humanitarianism seems increasingly a thin one to walk (Masiero and Bailur 2021), and too complex for some groups to appreciate, and thus negotiate or consent to.

As a matter of exemplification, consider the “introducer”. Introducers are those who are supposed to “mediate” between undocumented communities and central state administrations, personally knowing and being known by both,3However, under e.g. India’s Aadhaar, introducers only need to personally know and be known by the relevant state authority, i.e. the Registrar (Tiwari et al. 2022, 676). in order to “onboard” the former and provide previously unregistered individuals with a publicly trusted identity record. In other words, introducers are trusted parties that can guarantee for one’s *identity* – though they cannot provide for assurances so trustworthy as to unlock the granting of new *citizenships*. While identifying trustworthy introducers domestically is enough of a challenge already, with e.g. India and the Philippines facing more resistance to and mistrust in the role than expected (Adelmant, Bingham, and Cioffi 2023, 18; Jacob 2019, 16–17; Ramanathan 2015, 14), their mediating role along transnational chains of borderline identity patterns proves exceedingly controversial and perilous: Who qualifies for the role? How could it practically succeed? And who is liable in the event of failure (by fraud or incompetence)? These questions are inherently political, but rest on technical grounds insofar as they adopt the lexicon of “standards” to operate technology solutions that comply with legal requirements (or expectations thereof).

The complex interfaces between the introducer’s role, borderline patterns of citizenship and identity along migratory routes, and the technical embedding of compliance with norms on digital remote onboarding (if any), is the topic this paper seeks to explore and the contribution it endeavours to make. As the acquisition of people’s identities in borderline contexts is largely unregulated both domestically and internationally, particularly so far as the introducer’s role is concerned, this paper will aim at filling this policy space with meaningful recommendations. To do so, it will seek to address three interrelated research questions: (1) What should technical standards on remote digital identity onboarding ensure in borderline contexts? (2) How to ensure that the introducer’s function is enabled and supported by those standards? and (3) Why to caution against the involvement of unaccountable private actors towards the definition of the relevant policy problems and the selection of technical standards to address them? In fact, questions 1 and 3 are interrelated: no matter the needs one identifies in borderline contexts for technology solutions to address, private actors will filter (and dilute) those needs through their own interests. This matters here as technical standards, despite their public relevance for commerce and interoperability but also for legal values and concepts such as safety, dignity, or liability, are mostly drafted quantitatively (Infantino and Bussani 2023, 6) within private associations of engineers and computer scientists where democratically elected representatives and public servants are either absent or marginally incisive (Andersdotter and Olejnik 2021; Büthe and Mattli 2011). In terms of privacy, for instance, technical standardisation contributes to a privatised, US-centred institutionalisation of “outcomes by design” that turn ‘the meaning of privacy from a public and political issue enacted by judges, lawyers, and activists into a more technocratic’ exercise (Rommetveit and van Dijk 2022, 858) that is later transplanted into preemptive regulation without having qualitatively surveyed the relevant priorities and aspirations through democratically accountable, bottom-up participatory, context-informed, and user-sensitive regulatory channels.

This socio-legal work stands at the intersection of human security studies and international refugee law (or international human rights law more broadly). As for international security scholars, they have almost completely neglected discussions around technical standards and biometrics in contexts of borderline citizenship; when hints are made at these issues, it is from a broad criminological and anthropological perspective (e.g. Scheel 2019; Singler 2021; Tazzioli 2023) that certainly illuminates profound questions on human nature and institutional design, including group surveillance and algorithmic scrutiny as a form of political violence (Vecellio Segate 2022b, 62), but largely fails to specifically inspect ID-capturing devices *as a function of the problematic role of introducers*. When it comes to legal literature, instead, there is no paucity of sources on biometrics in migration control, the reasons why States implement these technologies, and the reasons why they are insidious from the standpoint of, for instance, privacy and non-discrimination laws (e.g. Casagran 2021; Díaz 2014; Thomas 2005). Legal scholars have discussed *ad abundantiam* – and mostly from a post-Marxist, biopolitics-of-exclusion perspective – the implications of biometric border control for e.g. stateless individuals and refugees (e.g. Nalbandian 2022), but they are yet to uncover *the connections among* the four aspects that I will be focusing on here: (1) the role of biometrics for remote identity onboarding as opposed to mere border control; (2) the design and enforcement of technical standards in borderline contexts; (3) the socio-legal challenges and limitations of implementing introducer-based onboarding systems; and (4) the controversial function of financial institutions (FIs) as enablers of these technologies and driving force behind their interoperable standardisation.

This is interdisciplinary work whose approach is mainly regulatory, sociolegal, and policy-oriented. The expert legal reader will find that a few elementary concepts (such as the distinction between “refugees” and “migrants”, or indeed the one above between nationality and citizenship) are briefly recalled: this is done deliberately, in order to accommodate the diversity of an interdisciplinary readership. While this qualitative research is primarily intended for and addressed to a legal-policy audience, technical standard-setters and other professionals (engineers, computer scientists, managers, auditors) on the more “quantitative” side of regulatory endeavours, as well as development scholars, humanitarian actors, and critical scholars of technology, will hopefully draw meaningful contextual insights from this effort, too.

The next sections will unfold as follows: Sections 2 and 3 will generally introduce the role of standards and the introducer as regulatory options towards trustworthy identity onboarding; Sections 4–7 will expand on each “borderline” situation separately, in order to outline their specific challenges vis-à-vis the introducer’s role and how standards may need skilled finetuning in order to address them; Section 8 will propose preliminary policy solutions to the highlighted issues, with specific reference to the three aforementioned research questions; Section 9 will conclude with an outlook on the warranted research ahead.

## 2 Standards’ Hegemonic Politics and Technical Attributes

Technical standards are supposed to foster interoperability and facilitate cross-border transactions and operations, including via commonly agreed protocols for the collection, storage, transfer, and processing of biometric data. The impact of standards on our daily life – and, in this case, on identity documenting – is such that their development should be entrusted with public regulators, while reality is that it has tacitly been outsourced to private standard-setting agencies that account to no one. As these agencies include some of the finest experts in the field and generally produce high-quality outputs, this situation is not necessarily to be regretted, though it does raise a few concerns on both a practical and a conceptual plane.

I will not dig too deep into this debate; suffice it to observe that developing countries – their engineers and, as a reflection, their concerns – often lament a sense of exclusion and neglect. Empirical studies which may prove or disprove these assertions are warranted, but it seems preliminarily fair to admit that technical standards still reflect both deeply rooted asymmetries in global knowledge production4On this concept, refer e.g. to (Castro Torres and Alburez-Gutierrez 2021). and what the European Commission phrases as a global struggle for technological hegemony (European External Action Service 2021). Building on an appearance of decentralisation (in that they are not directly issued or enforced by States), technical standards can be actually painted right at the core of complex webs of interlegality that shape what practices are internationally permitted and what are to be discarded (Cohen 2019, 202–237); considerations to this end are expressed in a lexicon of technical “necessity” or even “inevitability” but not infrequently shaped by cultural backgrounds and geoeconomic pursuits (especially between the US and China – Wu 2020, 107), with state-backed corporate commercial interests as value vectors and strategic drivers within data chain meta-regulatory efforts (Bloomfield 2012; Krisch 2005, 405; Levy 2008, 950; Nedergaard 2007). As I write, private entities keep playing an exceedingly problematic standard-setting role for identity applications and beyond, by actively engaging with policymakers and technical bodies towards the negotiation, socialisation, and legal “hardening” of standards they will be massively profiting from. I labeled their role as “problematic” not merely because private entities are by definition uninterested in catering for the public good as their primary objective, but more specifically because in these contexts, standard-setting work is relied upon by FIs that either ignore “borderline citizenship” situations or target individuals experiencing them with financial sanctions, on behalf of GN jurisdictions (most often the US).

As for biometrics, privacy-wise, the World Bank Group’s Identification for Development (ID4D) advises to implement standards ISO/IEC 27001 and ISO/IEC 29100.5See https://id4d.worldbank.org/id-biometrics-primer under the tag “How and where should biometric data be stored?” Most of the standard-drafting, however, is allocated to ISO/IEC Joint Technical Committee (JTC) 1 and its various subcommittees (World Bank Group 2017, 7–8). This JTC is focusing on credentials for user authentication, with an emphasis on security, interoperability, and robustness.6In plain language, “robustness” is the multidimensional ability of a computing system to deal with errors (failures, noise, erroneous training) in such a way as to preserve the integrity and validity of the output data (Ding, Janssen, and Crowcroft 2021, 165–166; Maple et al. 2021a, 10–12; 21). Many other variables, however, seem to be left to the periphery, including matters of cultural sophistication, data transfer, and free informed consent. Interfaces with artificial intelligence (AI) are also of concern, particularly if machine learning is deployed to validate profiles’ “alignment” with preset expectations and/or learn from a user’s patterns of engagement with the identity provider. In the hands of ill-intentioned agencies, algorithms might be trained to systematically discriminate fractions of the population, or to flag up individuals’ profile for further scrutiny by hostile authorities – in this event, individuals would not be prevented from onboarding; to the contrary, they would be even prioritised in order to be more rapidly “handled” (i.e. persecuted) by enemy factions, semi-privatised militias, or political opponents. Data patterns may end up feeding malevolent “risk profiling” that pursues identity onboarding for the only (or main) sake of censing (and censoring) regime challengers. Of course, not all such intents are malevolent: some may well adhere to genuine public-policy purposes, such as scaling-down the underground economy, preventing violent crime, or enhancing the safety of neglected neighborhoods; it all depends on the transparency and accountability record of those implementing AI solutions for biometrics. And when it comes to regulating AI applications, just like with any technology that impacts and records human movement, techno-specific regulation is not a panacea in isolation; ‘parallel efforts [are warranted] to set up a global institutional framework adequate to address the present and future global governance issues’ (Neuwirth 2024).

## 3 Mediating Trust Between the Centre and the Peripheries: Shall we Trust the Introducer?

Once a role devised by banks only, *the introducer* has recently captured the attention of public lawyers, policymakers, and sociologists for its application in the domain of technology-aided identity onboarding. For the sake of our discussion here, the introducer is defined as a mediating party that is trusted by both state authorities and identity-stripped individuals in order for the latter to prove who they are or to be provided with the required technical assistance to do so themselves. This second scenario (the introducer as a technology enabler and capacity builder) raises no substantial controversy: choosing introducers would still be challenging, but their role is fairly vicarious. Wider sets of controversies are raised, instead, by introducers who are entrusted to “validate” personal-acquaintance-based identity claims for all those the State should have already registered, because they indeed *are* citizens from a legal standpoint, but it failed to because of administrative, budgetary, political, and/or logistical constraints. In this scenario, the introducer bears an exceedingly cumbersome responsibility, which calls into question issues of cultural mediation, group belonging, reliability (at the intersection between trustworthiness and accuracy), and the potential for corruption.

Despite the ties they may have developed with their community, and despite (or precisely because of) their seniority, introducers might not have personally met their introducees in a long while. When that is the case, it seems important that the automated biometric identification systems (ABIS) the introducers avail themselves of can cater for e.g. voice recognition (Alharbi and Alshanbari 2023) or iris scans (Abdulrahman and Alhayani 2023, 2644) – in addition to facial recognition, or to the fingerprints which once represented the exclusive domain of automated fingerprint identification systems (AFIS). Fingerprints are severely inaccurate in contexts of poverty, lifelong manual labour, and “periphery” (so obviously typical of borderline situations) where, for instance, ‘[c]apturing fingerprints, especially of manual laborers, is a challenge […] because of the rough exterior of fingers caused by hard work’ (Ramanathan 2015, 11). There are also dozens of reported cases of migrants who prefer to burn their fingertips or dip them in acid as they are approached by border agents, or even as they start their journey via, say, the Mediterranean (e.g. Allen 2009; Feng, Jain, and Ross 2009; Grant and Domokos 2011; Krasny 2011; Merrick 2021); one may somewhat sympathise with the despair, atrocities, and fear-of-deportation that lead to their choice: whenever being known might equate to granting authorities a leeway to deport, remaining off-record is preferable at any cost (Reidy 2017; Trenholm 2016). Altering fingertips is not uncommon among non-migrant criminals, either (FBI 2015). Less relevantly but quite curiously, there are even people who are born without fingertips due to a genetic mutation (Groeger 2011; Sarfraz 2019). There is also advice issued against fingerprints hygiene-wise (Lodinová 2016, 96; Robbin 2022), to the extent that disinfection of appliances via UV-C technology is recommended – all the more so within or around refugee camps and assembly points, where epidemics of transmissible diseases are regrettably a constant. Granted: voice biometrics is not going to prove necessarily easier to collect (it arguably requires more voluntary input and more stable health conditions from the individual), hygienic/healthy for all parties involved (the next pandemics are forecasted as resulting from further coronaviruses), or univocal to match (tools’ sophistication in spotting falsified tones or misleading accents does not suffice yet, and concerns have been raised with regards to group discrimination – González Hautamäki et al. 2017; Jansen et al. 2021). And yet, fingerprints do not deserve to be deployed as the standard option, either: it really depends on the class of individuals to be onboarded, and on their expected degree of cooperation; in most borderline contexts, the degree of cooperation with the authorities (or with the introducer, for that matter) is not going to be helpful. Also, some new-generation systems of voice recognition via neuro-heuristics (Połap and Wozniak 2019) might displace issues of voluntary voice-sample provision – though admittedly raising a whole number of legal conundrums in relation to brain privacy, mental integrity, psychological continuity, and generally neurorights.

What I have just mentioned is not the only challenge in choosing the optimal combination between introducers’ profile and type of biometric data to be collected. Let me offer an example. One ID2020 pilot project in Indonesia sought to distribute fuel subsidies upon biometrics collection; the officials implementing the project noted ‘dust and dirt obstructing clean fingerprint reads, headscarves and veils worn by women that caused issues with facial recognition software, and network connectivity affecting collection and matching, especially in more rural and remote areas’, but they also remarked that ‘in regions where local government was more engaged in the project and in communicating with individuals, it received higher numbers of volunteers for participation’ (PEW 2020, 30). These passages arguably expound the paradoxes and contradictions of recruiting introducers to circumvent the just-mentioned obstacles. Indeed, suppose introducers were recruited by the Indonesian government: had they been sourced among local representatives, they could have abdicated to their formal duties (introducers are supposed to *mediate*, not to merely *represent* governmental agents themselves) and perhaps mistrusted by some “underground” citizens; and yet on the other hand, the program as a whole would have highly benefitted from local institutions as introducers, to the extent that mistrust, otherwise, shifts onto the other side (institutions would need to rely on non-governmental mediators). Indonesia also stands as an intriguing case-study for considerations around the relationship between population density, population size, and trust management. With reference to introducer-based onboarding, Pengiran (2012, 229) postulated that

[i]ntimacy captures how much of the targeted population is already known to the organisation. Having high levels of intimacy implies that the organisation can be more confident in making use of an introducer-based scheme, as it can easily support a transitive trust scheme that extends from known to unknown individuals.7All emphases removed.

This is extremely interesting, in that implications may be drawn from a demographic and error-rate perspectives. One could probably agree that intimacy increases with population density and decreases with population size: the bigger the population, the harder to establish meaningful connection, but the higher its density, the higher the chances that said connection can concretise nonetheless. These considerations bear momentous consequences for theorising about the feasibility and convenience of enabling large-scale introducer-based onboarding options.

Forthcoming onboardees are not the only class of potentially “underground” actors: introducers might well be so, too. Think about, for instance, taxi companies in South Africa: because of their job and the ties with local communities around the country, some drivers could well serve as introducers for digital onboarding, but because they themselves work in the informal economy (Genesis Analytics 2022, 11–12) and fear governmental auditing into the way they conduct their business, they would never come forward to serve as introducers unless the State pre-emptively stipulates relevant guarantees or tax amnesties – the latter would, however, apply only retroactively, and only to drivers up to a certain point in time, thus they might not prove as fitting the category’s interests as a whole. Similar limitations are acknowledged in the implementation of other digital technologies as well, including digital financial services – which would enhance inclusion, but are not trusted by informal workers who prefer cash-based transactions (Sharma and Díaz Andrade 2024, 9).

To capture the introducer’s function, I shall not neglect some deeper sociological implications of its recruitment and deployment, that rest with collective fears and the public order. One question border officials are often displeased about, and that has a bearing towards the very role of the introducer, concerns whether ethnic self-identification reduces or weakens national self-identification, with corollary quandaries around the impact of such disclosures and identifications on social stability and the security of border areas (e.g. Luo 2022, 93). For our purposes here, one consequent question might be: should the introducer promise to the “introducees” that their self-identification will be prioritised and featured into their identity documents? In the positive, could different technical standards be envisioned to cater for the different expectations and customs of different ethnic components being registered? This is a paradigmatic illustration of how *nationality* as cultural stratification matters even when one, legally speaking, is only deploying technology to address *citizenship* (or more generally identity) issues.

In any case, the introducer should behave as neutrally as possible. In particular, it should not be perceived as “siding” with the authorities to “assess” one’s worthiness for citizenship and identity recording. In legal sociology literature, “good citizenship” is defined as all those attributes that mark one’s good standing towards a country’s rulers (Sköld 2019, 218); it comes as exceedingly problematic whenever legal citizenship is offered in conjunction with “credit score” systems linked to one’s “prosocial behavior” – which may easily turn into subservience and obedience and foster chilling effects on decentralised traditions and belonging. Furthermore, tracking for the purpose of assessing one’s “good character” should be disallowed, and potentially replaced with “group credentials” (as e.g. confirmed or disproved by diasporas) as pledged by the introducer. Too many States still deploy “good character” arguments to decide against naturalising stateless individuals (Mrekajova 2012, 35–36), often based on criminal precedents of dubious momentousness, fairness, and relevance, whose record might be even oral only and/or referred to several decades earlier. In fact, good-character tracking may dissuade stateless individuals from seeking official recognition and identity onboarding. Statelessness is also originated when a State creates a legal basis for factual denationalisation that denies the repatriation of citizens for alleged “terrorist” (or most often, terrorism-supportive broadly defined) activities abroad (Tripković 2023).

As all “borderline citizens” – and especially stateless persons – are particularly vulnerable psychologically and exposed to the risk of psychiatric traumas and poor mental-health conditions (Herberholz 2022), the introducer may also be valuable in assisting all those who appear confused or inconsistent in retracing their roots or self-identifying (individually or community-wise).

For the time being, the introducer role should be dissected into the entire spectrum of its policy, economic, and sociolegal implications, standing clear of hyper-enthusiastic conclusions. Issues of trust, corruption, and traceability abound, and nowhere in the world an introducer-based system has ever been tested successfully on a large scale (Belorgey and Jaffrelot 2021, 17–18; Sathe 2014, 100–101). Even in India, where introducer-based enrollment was enacted to cater for the non-universality of citizen identification (Pengiran 2012, 228), Aadhaar’s data reports that only the 0.3 % of subscribers availed themselves of the “introducer” route: all others simply joined the scheme to “update” or “duplicate” paper-based identity credentials they already possessed (Access Now 2018, 14–15; Privacy International 2017, 22). This is all the more evident if one considers that Aadhaar provides no citizenship certificate: it is merely an identity matcher which does *not* alter the legal status of the onboardee (Nilekani 2013) – which is thus going to be proven with paper documents, still. To say it all, Rao (2019, 18) hypothesised that *precisely because of* the relatively low trustworthiness of introducer-based enrollment, Indian authorities decided to forego the linking of Aadhaar onboarding with new proofs of status. Regrettably, despite this policy backtracking, relevant fractions of Indian rural society enrolled into Aadhaar upon convincement that it would have been helpful and indeed legally required for them to secure proof of citizenship status for their children (Bhatia, Donger, and Bhabha 2021).

## 4 Borderline Citizenship, First Scenario: International Refuge

Individuals have the right to flee from persecution and misery (albeit some authoritarian regimes tend to ignore it: Vecellio Segate 2021, 223), and States are under an obligation to process their substantiated claims of asylum, which if approved would turn them into proper “refugees”. While no destination country has an absolute obligation to admit individuals (and especially asylum seekers whose application for refugee status is pending), States do have an obligation to *non-refoul* them to unsafe destinations where they would probably endure harm and indeed persecution. As complex as it might sound, this is what international law dictates: no obligation to admit, but obligation to non-relocate to unsafe harbors. If States do decide to relocate asylum seekers to destinations they consider “safe”, legal implications abound (Foster 2007), not least when it comes to identification, personal data, and information exchange with “partner States” – and technical standards and protocols applicable thereto.

As States nowadays tend to criminalise most informal, unofficial migration flows, asylum seekers perceive the possession of identity documents as counterproductive: the more univocal and established their identity, the higher the chances they will be repatriated against their will, or that authorities between the pushing and pulling countries will liaise about their background and fate. This liaising, in particular, defeats the very purpose of having an international refugee law framework in place, whereby asylum seekers can seek protection for the persecution they experienced in their country of departure. Migrants are on the move to build a new life elsewhere, or simply because moving has always been an intrinsically constitutive aspiration (and arguably an inalienable right) for any human being; asylum seekers, on top on it, might be fleeing for escaping a regime that discriminates them and violates their dignity. Hence, the number of ID-free migrants who reach the shores of countries like Turkey, Italy, Spain, or Thailand is frankly unsurprising. ID capture at borders, especially through biometrics-based AI systems, largely fails to account for these individuals as humans with their own needs, potential, and aspirations: rather, they tend to assume they represent a security threat or, at very best, a burden to techno-manage (La Fors and Meissner 2022).

One could be instinctively tempted to argue that the United Nations High Commissioner for Refugees (UNHCR) should strive to gather and share with the authorities as extensively and accurate identity information as possible, in order for States to process claims of asylum as rapidly and comprehensively as they can. On-the-ground reality, however, speaks another truth. In fact, UNHCR personnel should *not* automatically share these identities for good. If they did, as it regrettably occurred a few times already, how could asylum seekers trust this process, and particularly that States will not attempt at forcedly gathering or misappropriating information databases possessed by the UNHCR (not least under the guise of prosecutorial investigations arranged ad hoc)? In fact, even after asylum seekers’ claims are approved and they are granted refugee status in their new country, States of origin will hardly fail to put pressure on international organisations (IOs) to disclose refugees’ data, for either persecutory or, more neutrally, statistical purposes: state administrations retain a granular interest in knowing exactly who fled and why, and they are going to petition the UNHCR and similar organisations to release such data. Providing States of origin with refugees’ identity data would, however, defeat the very purpose of international refugee law, as a global system of last-resort protection for individuals who are persecuted (or perceive themselves to be at risk of persecution) in their original country of citizenship and/or habitual residence. The UNHCR has been sharing asylum seekers’ data with the potential host State for decades, without any consent from these individuals nor any mitigation framework in place; for instance, the US Department of Homeland Security (DHS 2019, 14) notes that

DHS and UNHCR have been indirectly sharing biographic information during the refugee resettlement process for many years. The MOU between DHS and UNHCR for Refugees on the Sharing of Personal Data expands that information sharing to include biometrics.8Emphasis removed.

What this means is that their data has been shared without consent, but also without a number of safeguards related to, for instance, cybersecurity. No technical standards applied mandatorily to these data transfers, nor were the transfers themselves recorded anywhere public. As if this were not serious enough, in several instances the UNHCR (and similar agencies) has been succumbing to political pressure to share data *with persecuting governments from the States of origin*, too – not least during the infamous Rohingya crisis (Holloway and Lough 2021; Human Rights Watch 2021; Rahman 2021). This is a blatant violation of international law, and exposes the need to design identity onboarding procedures that build on solid laws and equally solid technical standards enabling their meaningful enforcement – especially when State-participated IOs are involved. Distributed ledgers and open registries are warranted, to ensure that not only IOs refrain from disclosing this data directly, but that host States do not attempt at doing so themselves with authorities in sending countries, at a later stage. This happens frequently owing to “diplomatic comity” but also in the hope to stop migrations *ab initio* – which, again, defeats the very purpose of having an international legal framework for asylum seekers. Sharing UNHCR aggregate data, instead, raises in principle no legal issue, and it might support political science researchers (e.g. Marbach 2018) – though again, the extent to which “aggregate” data is unserviceable for States of origin to reidentify (and keep persecuting) single individuals would deserve inspection on its own, as well as formalisation into appropriate technical standards for data sharing.

One may further wonder *what* identity the UNHCR is supposed to collect (and expected to share) exactly. If that is refugees’ original identity, i.e. the one they were known in their country of origin by, then perhaps an introducer will be needed, who is proficient in both languages and can testify to their identity. Risks of corruption and fraud abound, as do the chances that undercover governmental agents, private security contractors, or cover smugglers propose themselves for the role.

When, instead, refugees are (temporarily?) assigned a new identity, an introducer might still be of aid to “prove” family ties and, with them, verify claims of inheritance, property, forced displacement, war damages, parenthood, and the like. In this event, how to ensure that previous identities are fully erased? To exemplify, what if the “receiving” government comes into possession of pictures reporting the previous identity of the refugee, eventually linking the latter to their personal history and issuing restricting administrative decisions based thereupon? As open-source information (and indeed pictures) online is on the rise, the risk cannot be underestimated. And how to trust that such government is not going to misuse such information, e.g. in the context of regime collapse?

These are all circumstances that rely on technology solutions to enhance trust and indeed provide “trustworthiness”, in compliance with technical standards that shall be designed to cater for the interests of the most vulnerable. For instance, right upon a *coup d’état*, one of the immediate measures adopted by the prevailing party is that of militarising and most often fencing and locking the country’s border,9To exemplify, this is what happened most recently in Niger (France24 2023); given Niger’s centrality in Sub-Saharan migration patterns towards North Africa and eventually Mediterranean Europe (Financial Times 2023; Vecellio Segate 2015), this case is of broader significance here. On the deployment of biometrics technology for migration management purposes in Niger, see (Dauchy 2022). while the previous ruling élite is either arrested or sent into exile: how to ensure, then, that identity information is not used against the dethronised to target them and their supporters/voters domestically while preventing them from fleeing to and seeking refuge in neighboring countries? And even if they fled successfully, how would those neighboring countries behave data-wise in terms of formal and informal information sharing with the new rulers? Technology solutions should anticipate and possibly prevent all such problems. For instance, should identity onboarding data be exclusively accessible to certain institutions or to each ruling élite only until power is handed over to the next one and all data expired with it? A solution whereby population’s data is only accessible to a ruling party while the next one would need to restart from scratch is counterproductive and unlikely to be accepted; however, in highly unstable regions and insofar as vulnerable or especially “exposed” segments of the population are concerned, one could devise a system of pre-emptive and voluntary protection whereby specific individuals could opt-out from the identity system should regime change materialise – either temporarily, or until they are able to flee and receive credible assurances from the newly installed rulers.

Either way, i.e. the refugee keeping their previous identity or embracing a new one, identity duplication is of aid to no party, as the person could then “free ride” on the two identities to escape regulatory reach from both the “original” and the “subsequent” regulators, governments, administrations, and courts. Also, from a law-enforcement standpoint, identity duplication greatly enhances the likelihood that transborder criminals are caught later than they would have been, had they been endowed with just one identity – at least, *one at a time*.10These considerations are valid for refugees only, but might not be as applicable to or suitable for “non-borderline” situations. Quite the opposite is true: purpose-limited multiplicity of digital identities (which does not necessarily escalate, however, to the level of duplicated identities) might help foster users’ privacy by disrupting cycles of patterns extractions and data exhaustion. Refer e.g. to (Access Now 2020, 4). In borderline contexts, identity duplication is also to be prevented in order to defeat the “ghost beneficiaries” phenomenon associated with shelter and aid provision across refugee camps and similar arrangements. I acknowledge, however, that from the standpoint of humanitarian organisations it is in fact timesaving and operationally convenient to accept that individuals might be recorded differently by different NGOs, so long as they are univocally identified by each of them throughout time (Schoemaker et al. 2021, 18). Technically, these are short-term practices that should not be officially endorsed, but they can be justified as grounded in regulatory shortcomings that make it unclear who should identify individuals in “borderline” situations, what the applicable procedure is, and what is the legal basis for other organisations to trust the identity assigned by another organisation. In other words, there is currently no legal basis for humanitarian actors to take any identity assigned by other NGOs as definitive and trustworthy – hence, they often resolve to assign such people yet another transitional and “NGO-specific” identity.

While *concomitant* non-duplication would be essential, multiple identities *at different times*, while definitely an additional hurdle in terms of e.g. transnational crime investigations, can prove life-saving against backdrops of persecution and refuge. It is often reported in scholarship that through *universality* and *intimacy*, governments endeavour to secure authenticity, while uniqueness is secured through *obligations* and *performance*. I will spend some more words on authenticity requirements, but uniqueness, too, is especially challenging in contexts of refuge, while being too often disregarded in biometrics literature. Scholars generally posit that ‘[o]rganisations’ desire for uniqueness is driven by concerns of identity fraud, where individuals might attempt to enrol multiple times, potentially using multiple personas, to gain extra benefits’ (Rahaman and Sasse 2012, 42), and while this is indeed a concern, the other side of the issue is virtually always neglected. I am referring to contexts whereby it is convenient for governmental, para-governmental, or even non-governmental organisations to have *multiple personas registered under the same denomination*. This reads counterintuitive, but an example should hopefully clarify the matter. Suppose you are a refugee, fleeing from political persecution; suppose, further, that customary traditions in your rural village demand denominations (what one understands as “name and surname”) be inherited vertically across generations with little to no variation, in order to remark one’s belonging and tribal affiliation. Conjecture, then, that this tribe-based denomination makes it easier for your persecutors to target your extended family members not only in practice but also *de iure* (e.g. by reducing pro capita welfare benefits to a few members only, based on homonymy, or worse even, celebrating trials under summary justice proceedings, or pretending to “mistake” one member for another). In all such contexts, onboarding should be designed in such a way as to secure not only that one individual registers multiple accounts, but also that several identities do not get conflated under the same definition because of (genuine or fabricated) instances of tribal co-denomination. This helps ensure that a remaining family member is not targeted in lieu of a similarly named family member who escaped (or tried to) the relevant jurisdiction to petition as a refugee abroad.

Either way, transitionally, potential contacts of the refugee in the destination country can prove of assistance for trusted recognition – of the refugee’s previous identity if they keep it, or of e.g. family ties if they wanted to embrace a new one (think of all those – especially women – who change their surname when establishing themselves in a new country). Provided that their ties with the destination country are lasting and “clean” enough, a selection of these potential contacts could serve as introducers, but they might not agree on being remotely identified: some of them might prefer to be identified by authorities on-spot, in-person, through traditional paper documentation, without releasing their records to a newly conceived centralised system – especially if AI-powered. State authorities, along with the system’s designer, shall make sure that introducer identification never becomes a disincentive for destination-country contacts to come forward and help whenever they can, also thanks to their ties to local groups from relevant diasporas.

The erasure of refugees’ previous identity raises a host of issues in its own right. To begin with, how could they claim back their financial assets deposited within traditional bank circuits under the previous identity? Are state laws flexible enough to accommodate a combination of biometric authentications as opposed to ID-based recognition systems, even for the most complex operations to be performed in person? And will the management of one’s identity by banks impact the erasure (or lack thereof) of the same person’s identity by state authorities? Policy reports on how to develop technical systems for digital identities that comply with anti-money laundering regulations, confirm that governments back (or even themselves implement) limited-purpose ID systems in addition to general-purpose ones (Financial Action Task Force 2019, §52–53), and that ‘[i]n the digital era, we have begun to see new models, with digital credentials provided by, or in partnership with, the private sector being recognised by the government as official proof of identity in an online environment’ (ibid., §54). Banks are thus indispensable actors in identity management with public implications, which makes it even more alarming that some of the most updated guidelines on these matters (e.g. European Banking Authority 2022) provide no satisfactory answer to the questions posed above – no mention of identity reassignment, identity erasure, liaison with different state authorities, and so forth.

From a slightly different angle, it was accepted that ‘[i]n the case of refugees, proof of official identity may also be provided by an internationally recognised organisation with such mandate’ (ibid., §55): the interfaces between refugee law and public-private partnerships (PPPs) in this respect are yet to be explored. For example, would most States be prepared to endorse an alternative banking circuit dedicated to refugees, supported by its own technical standards, whereby refugees could safely declare their “previous” identity for the purpose of claiming their savings back, to then move on with their new life and new comprehensive (can even be general-purpose) identity? Shadow banks already exist and work well, but alternative *official* circuits would be unprecedented.

To be sure, my suggestion is not that asylum seekers shall express informed consent and enjoy a right to non-disclosure or non-registration of their identity: this would not be practicable, and it would not be supported by customary law; rather, what I advise is that asylum seekers do have an obligation to undergo identification, so long as States formally pledge to never deploy it against them for as long as their vulnerable condition endures – especially towards forcible repatriation. I accept that finding the “right” balance is an edging exercise at a constant risk of failure due to States’ political overreaction to what they perceive as migratory “threats”, mostly motivated by domestic deals with the electorate. To put it straight, political overstretching is always round the corner – paradoxically, especially so in democracies whereby politicians are accountable to electorates’ sentiment.

The already mentioned WB’s ID4D suggests that in the case of refugees, the UNHCR itself could serve as the introducer (World Bank Group 2022, 2). This is idyllic advice as it is naïve: it would probably stand as the cheapest option, but for a host of political and practical reasons, States are unlikely to take it on board. The considerations above should read explanatory enough, but I wish to illustrate three reasons more systematically. First, international humanitarian officers are not directly accountable to any State where they operate, meaning that their liability and subjection to prosecution for e.g. willingly providing false information – or accepting bribes – are unclear. Second, just like in many other sectors, humanitarian appointments tend to be shorter and shorter, due to generational job casualisation but also increased mobility, project fragmentation, overreliance on private philanthropy, and funding uncertainty; this means that controversies or follow-ups are not necessarily viable from a practical standpoint. Third, and perhaps most saliently, humanitarian personnel faces increasing political backlash – when not wholesale criminalisation – Ferstman (2019) by the entire political spectrum of most “developed” countries, and especially by their right-wing (or conservative) parties, who are concerned about the State losing grip over its borders and on retaining the ultimate decision over a person’s admission into its territory. In between apathy and desuetude (Gleichert 2020), the Refugee Convention and corollary obligations (for those States which have undertaken them) are the subject of long-standing controversy, to the extent that it appears unlikely that UNHCR officers alone could guarantee for refugee’s identity without the involvement of key individuals (not necessarily governmental ones!) from the “sending country” – and the “pulling country” alike.

As I will expound *infra* for other “borderline citizens” just as much, one of the challenges then is to persuade third parties to “take the risk” of aiding a refugee’s remote onboarding without assurances that such parties (i.e. the introducers) would not be registered as well. The reasons why introducers would prefer to go unrecorded are several and perhaps mostly obvious: if they are summoned by the pulling country, they risk being identified as “immigration aiders/supporters” or even face detention risks for complicity (e.g. “aiding and abetting”) with illegal migration schemes; if, instead, they come from the sending country (or from within the same region), they might not want to risk their own immigration standing in the new country, and/or they might be escaping from the same source of persecution – that will hopefully renounce to extending its reach over them, if they go unnoticed for long enough and have left no family members back there.

## 5 Borderline Citizenship, Second Scenario: Internal Displacement

Internally displaced persons (IDPs) are those who are forced to leave their habitual place of residency but are yet to cross an international border. Notably, due to the vastness of some of the most politically unstable and environmentally exploited countries (think about the DRC, Niger, Angola, Libya, Argentina, Sudan, Myanmar, Algeria, …), being internally displaced might push individuals farther from home than many cross-border migrants would be.

Statistics on IDPs are unstraightforward to read, as displacement depends on extemporary causes that may unleash *but also resolve* relatively fast, so that the need ensues to distinguish between on-average yearly IDPs and IDPs at any given time. While a long-standing target for sociologists and anthropologists, IDPs have garnered the attention of public international lawyers and political scientists only relatively recently (Dirikgil 2022; Draper 2021; Orendain and Djalante 2021). Aside from the non-binding UN Guiding Principles dedicated thereto, there is no detailed engagement with IDPs’ specific needs – let alone rights. As for the present paper, if these people are displaced *internally*, i.e. domestically, then why should we extend our analysis on borderline phenomena to them? I reckon there are at least three sound reasons to do so.

First, as a follow-up to their displacement, IDPs might easily decide or find themselves forced to themselves become asylum seekers, nomads, or even stateless individuals. This occurs most frequently through forced displacement, which is why a positive obligation is bearing on States (for instance under the Palermo Protocol) to ensure that internal displacement does not turn into human trafficking, and were this regrettably to happen, that those affected enjoy protection of their privacy *as a form of protection of their identity* (Human Rights Council 2023, §58).

The second reason is that internal displacement is set to increase dramatically in decades to come, mostly owing to climate change, corporate pollution, ecosystem collapse, fragile and failed States, unplanned overurbanisation, and conflicts over resource access, ownership, and distribution. Legal and policy classifications based on ‘hazards, exposure, vulnerability, and capacity’ (Cantor 2023, 12) will need to adapt to on-the-ground realities whereby local institutions’ failures in protecting their territories and people will translate into civic detachment and trust withdrawal from local communities. This makes it essential to foster preparedness on the identity side, too, all the more considering that when paper documents go missing or irreparably damaged, local administrators who typically serve as introducers might well have fallen victims to the very same disasters that displaced those in need of identity onboarding.

The opposite side of the argument – and the third reason – is that those very same local administrators are precisely those who most probably contributed to (or failed to prevent, also out of bribed connivence) the disasters which caused the displacement; hence, one would not want them their maladministration to administer IDPs’ identity (re-)onboarding!

Of course, when it comes to IDPs, the problem to be addressed is not their first identity onboarding, but rather the combination of: (1) documents going missing or destroyed in disasters, and looting/misappropriation thereof, or fraudulent claims of having lost or been deprived of them; (2) deterioration of data infrastructure and disruption of data services that were used *inter alia* for identity onboarding; (3) reconfiguration of on-the-ground geometries of ethnical affiliation and community belonging, which might impact the availability, profile (i.e. recruiting criteria), care, and/or trustworthiness of introducers.

## 6 Borderline Citizenship, Third Scenario: Stateless Individuals

Statelessness is a particularly disadvantageous condition for individuals, as they will be placed outside the protective scope of any domestic legal regime, lack access to welfare benefits, and be denied civil-political rights, along with property entitlements and access to their own saving and accounts (La Lumia 2022). It is also dangerous, as it places them under threat by criminal actors and law enforcers alike: stateless individuals can neither denounce abuse by authorities, nor expect the latter to protect them from criminality. More accurately, they may well denounce abuse and crime, but the follow-up is probably going to prove inattentive and ineffective. This is why, as remarked by Kingston (2019, 57),

[s]tatelessness is recognized not only as a violation of the “right to a nationality” but also as a root cause of additional rights abuses. […] Legal status is only one step in a long journey toward full rights protection; statelessness is both a cause of marginalization and a symptom of it. That is, most stateless populations lack legal nationality because they face systematic discrimination from the beginning.

This reads rather obvious to anyone who has perused statelessness statistics: the vast majority of stateless individuals was born in conflict areas, failed/fragile States, or contended territories. They might have preferred to relinquish their citizenship to escape persecution, as an alternative to applying as refugees; or they might not have been granted citizenship at birth by hostile and/or incapable regimes.

The most pressing statelessness-related emergency is faced by Sub-Saharan African countries, where large segments of the population are totally unrecorded; the relevancy for statelessness is that when these individuals submit petitions to regularise their position and be officially censed, they are not issued any proof of identity, let alone one they can travel internationally with. While they are technically citizens of their country, they cannot prove it to its authorities, nor to any authorities abroad; hence, they live as *de facto* stateless individuals (Institute on Statelessness and Inclusion 2017, 26). *De facto* statelessness is indeed the condition of those who are citizens according to the law of at least one State, but whose citizenship is not recognised or declared by any documents possessed by such individuals (Massey 2010; Sawyer 2011).11For a couple of (minority, but representative) dissonant voices, check (The Equal Rights Trust 2010, 52–84; Tucker 2014).


Nonetheless, it is not a “developing world”-problem only. In Canada, for instance, several stateless persons face removal-aimed indefinite detention, with authorities being unable to either regularise their domestic status or justify their deportation to any specific safe *and relevant* place; this obviously takes a toll on these persons’ mental and physical health, let alone the capacity to plan anything close to a future (Kane 2019). Similar stories are shared by stateless individuals in the US, with each administration renewing their promise to legislate in the area but eventually failing to fulfil it (Ambartsoumian-Clough 2022). In the EU plus Norway alone, in 2018, there were nearly 400,000 stateless individuals (European Commission 2021, 1), and the UK is one but a few States in the European macroregion to have established a procedure for processing statelessness claims (Bianchini 2017, 86); this requires the claimant to have exhausted all applicable good-faith efforts to prove their identity and petition for relevant citizenships. As yet another example, Belarusian citizens living abroad become stateless when Belarus fails to renew their passports remotely while factually forcing them to stay abroad as they are persecuted by the authorities (Kolchyna 2023).

Although a few States understand stateless individuals as lacking proof of citizenship, international law defines them more narrowly as lacking citizenship altogether, either because they were stripped of it by their State, or because they relinquished it themselves (Council of Europe 2021, 9).12Note that this source employed inaccurate terminology: “citizenship” should have been preferred in lieu of “nationality”. In fact, the main challenge here is that statelessness is often an informed, voluntary choice; this prompts ethical considerations around the suitability of deploying identity management systems for deliberately recording stateless individuals. Careful however that even they might long for an identity, and indeed have the right to acquire one: identity and citizenship are not necessarily coincident, meaning that one State might grant an individual an identity without making them a citizen. The 1954 Convention relating to the Status of Stateless Persons, which despite the low ratification rate is widely considered customary law and as such binding on all States regardless of membership, declares at Article 27 that a stateless individual enjoys the right to identity documents (ibid., 3–4). This raises dilemmas as to the nature of such documents and their bureaucratic “interoperability” for several purposes (transnational criminal investigations, transnational tax governance, but also mere travelling): if they are not passports strictly understood, why should other States recognise them? Would that mean that such granted identity, too, is confined to the State granting it with all others not upholding it? On the one hand, the same Convention at Articles 25 and 28 enshrines the right to travel documents for stateless persons who have a residence permit in the host state, as well as their right to administrative assistance of the type that other foreigners would normally be able to obtain from the State of their citizenship, but this is factually harder even if strengthened by Article 6, pursuant to which no requirements can be imposed on stateless persons which they cannot comply with due to being stateless (ibid., 5). While doctrinally sound on paper, these obligations, when it comes to travelling, are only meaningful insofar as the documents granted by the host State under the Convention are granted recognition by the State these individuals intend to travel to. Given the narrow (albeit expanding) subscription base of this Convention (van Waas 2014, 13–14), even assuming that the host State is a signatory, in practice it is unlikely that the destination State will be a signatory as well. Major sending and receiving jurisdictions like Indonesia or India have never ratified the Convention, which is factually ineffective when it comes to cross-border movement. Given that no State is *a priori* responsible for granting identity documents to a stateless person (ibid., 9), physical presence within a State’s territory is the only way for said person to expect such State’s authorities to release documents – at least on public security grounds; however, international travel remains advised against, therefore there is no need to account for it towards the biometrics infrastructure that enables the identification of stateless individuals.

Another applicable treaty is the 1961 Convention on the Reduction of Statelessness, whose claimed status as international customary law is far less persuasive. These two statelessness-specific treaties are complemented by “general” human rights conventions dedicated *inter alia* to non-discrimination, disability, and childhood.13For a model questionnaire (sort of a checklist) to support the identification of stateless children, see (European Network on Statelessness 2023).


States are arguably bound by international law to refrain from enacting laws that may foster legal or factual statelessness (Mohsin 2020, 5), but whether they are under an obligation to progressively reduce the root causes thereof is less settled; this marks the unlikelihood to witness a rapid political resolution of this global issue. The obligation to refrain from producing new statelessness is bearing upon States not only in times of peace, but also (and especially) in conflict scenarios. Indeed, under international humanitarian law (IHL), States shall uphold their duty of care when exercising *de facto* control over a territory and its population (ICRC 1987, §2.5b). This means *inter alia* that stateless individuals shall be able to access redress (Recalde-Vela 2019); for this to happen, possessing a (citizenship-unlinked?) identity seems a prerequisite. Authoritative adjudicators (e.g. Court of Justice of the European Union 2014, §21) increasingly hold that at least some of these obligations extend well beyond the formal IHL remit, as to encompass for instance hybrid domestic conflicts as well – which are more and more common.

Meanwhile, on the technical side, outlining the legal demarcation between statelessness and other more commonly known forms of “borderline citizenship” will hopefully clarify how technical standards should be designed to accommodate this phenomenon. To begin with, any system should incorporate a function to ask these individuals *whether* they would like their identity to be recorded: while they have the right to apply for an identity to be recognised by the relevant State, no obligation is bestowed upon them to do so. Stretching the argument formalistically, the State might still decide to somehow record the existence of such individuals and to do so, they will need to identify them in some way, but said identification does not need to be undersigned or “accepted” by stateless persons. This distinction is sophisticated but not merely formalistic: it might matter for instance towards one’s degree of civil or even criminal liability in the courtroom.

Whether bilateral or not, data gathering should take place at least in an aggregate form, in order for the phenomenon to be statically assessed more accurately. As of today, no statistics seem fully reliable, because they are either based on voluntary self-disclosure, or based on incomplete primary data such as birth registries in the “Global South”. Also drawing on recommendations from the EGRIS Technical Progress Report submitted to the Fifty-third meeting of the UN Statistical Commission, more work is warranted towards demographic modelling and statistic data linkages, and the scientific capture of their sociolegal implications (Strode and Khanna 2021).

A cloud-based open registry, possibly in the form of a blockchain (Gstrein and Kochenov 2020; Hobson et al. 2023), should be established and entrusted to an independent organisation, possibly funded by wealthy diaspora networks as opposed to reliant on voluntary state contributions, to be placed under the nominal authority of a specialised IO. Because States are active producers of statelessness and their bureaucratic apparatuses cannot necessarily be trusted, the registry should be accessible to them but entries should be modifiable by stateless individuals themselves only, in such a way that while voluntary statelessness might fall off-radar, most involuntary one is consistently recorded and each case can be brought to the attention of a selected group of States to be selected upon predefined criteria.

To break the intergenerational cycle of poverty and misery rooted in statelessness, special focus is called upon recording new births whenever feasible (Human Rights Council 2014, §9–70; OSCE 2017, 31; 51): no matter the parents’ legal status, and possibly irrespective of the latter’s consent, newborns are to be provided with identity and citizenship as soon as possible – well-founded parental concerns around security (to be judicially appraised) representing the only exception.

## 7 Borderline Citizenship, Fourth Scenario: Nomadism

### 7.1 Traditional Nomadism

Traditional (i.e. “periodic” and “on-the-road”) nomadic movements are as old as humanity itself. Today, uncontacted and unmapped populations aside, pastoral and peripatetic itinerants mainly traverse the Sahel, Maghreb, and Mashreq regions, with minor manifestations in Central and Latin America as well as Central and South-East Asia, Iran, and some Pacific archipelagos. They pose challenging security dilemmas for the jurisdictions concerned, for the protection both *of* and *from* nomadic individuals; their univocal identification could perhaps help reduce these inconveniences. To address these peculiar migrations from an identity-onboarding standpoint, one may conceive of infrastructure that either “follows” the nomads or waits for them at regular intervals – needless to remark, the second solution presupposes that nomadic movements repeat themselves cyclically and adhering to the same seasonal patterns as well as routes. The role of meta-introducers here could be that of keeping governments informed about expected deviations of forthcoming nomadic movements from the expected routes and seasonal periodic patterns.

In the first case (“following” the nomads), one shall prefer mobile devices, while in the second case identification stations should be permanently placed along the way. Data-wise, either solution is fraught with jurisdictional apportionment issues. Provided that nomads’ identification is understood as a form of supranational collaboration between impacted countries, the data infrastructure that underpins said stations can be thought of as international territory, no matter where it is located – just like an airport. A regional community of States would officially recognise the infrastructure and endow it with some form of transnational legal status. Shared infrastructure relies, in turn, on shared standards for data transfer: be it a mobile app (e.g. Rios 2023) or a permanent station, the solution should incorporate one function for domestic data processing and another function for data transfer across all those jurisdictions that joined the remote onboarding project. Any transfer scales up the likelihood of data breaches and data leakages, so that only the “minimum core” should be shared among all parties; this is essential, as jurisdictions globally still tend to dismiss the momentousness of data breaches as a *public* normative issue (Vecellio Segate 2020). Further, as these nomadic movements can take time, one shall make sure to capture e.g. biometrics whose validity does not decay with aging, and to re-check for newborns or deceased on nomads’ way back.

This paper is mainly concerned with cross-border phenomena, including cross-border nomadic movements, but some of the challenges in technologically recruiting and supporting potential introducers in nomadic contexts can be exemplified at even the simplest level of domestic nomadism: homelessness. In India, when local NGOs started to act as introducers and sought to onboard homeless individuals, they realised (Rao and Greenleaf 2013, 294) that their effort

had become an advantage for more stationary homeless people with permanent sleeping places. The sedentary citizens of Delhi’s streets proceeded to gain a new identity that resulted from negotiations between bodies and machines. Yet more mobile populations either missed the survey or could not be traced after they had left the recorded location. Enrolment proved to be difficult.

Even domestically, it is (unpredictable) mobility that makes it challenging to recruit appropriate introducers. Introducers themselves, from a legal liability standpoint, grow increasingly anxious about introducing thousands of individuals they might have barely met once, and whose conduct and intentions they feel they cannot genuinely vouch for – both prior to the process and upon successful introduction (Singh and Jackson 2021, 11–12). Releasing introducers from liability would likely boost the risk of corruption, but the current state of affairs obviously defeats the entire (declared) purpose of Aadhar, built on inclusion (Dixon 2017, 547–548; Sen 2019, 2). How could technical standards soften this tension? First, they could be devised to enable identity validation from previously enrolled homeless individuals from the same areas: mobile homeless people tend to share information about itineraries and, with that, about where they come from and potentially who they are. This might help enroll subsequent people from the same communities. Second, in order to rebalance introducers’ own liability, the onboardees should accept the burden of having their movements tracked over a “probationary phase”, to make sure that their reported habits and roots are more trustworthy. Having these trajectories and narratives matched by human operators on a very large scale would probably overburden the system, so that algorithms should be designed to map narrated trajectories and match them with the actual habits of the onboardees during probation as retrieved from the tracking technology. Of course, these procedures should remain voluntary for any homeless individual, and exclusively aimed at making remote identity onboarding more transparent and efficient for all parties.

### 7.2 Digital Nomadism

Ignited by the COVID-19’s pandemic lifestyle, digital nomadism is normalising even in reportedly remote and Internet-poor lands such as Papua New Guinea. Digital nomads are not too much of a concern for public authorities, in that they live on their own money and theoretically “contribute to the economy” of the place where they decide to (temporarily) settle and work remotely from. This is precisely why most Global North countries are racing to attract this nomadism, not least through “golden visas” and tax breaks, and codify the legal frameworks applicable thereto. However, as the phenomenon is set to grow steadily at all latitudes, contentions will soon arise on how to apportion the income of such individuals, just like debates have unfolded for decades (and not settled yet) on how to apportion the profits of multinational corporations with all their subsidiaries operating worldwide. To that end, federated protocols in income-tracking standards might prove of assistance:14More on federated standards at (World Bank Group 2017, 5–6). each State could upload their taxed-at-source income related to an individual, so that other States could both be aware of such income and avoid to double-tax it.

In turn, the corporations that digital nomads work for should agree on a technical standard that enables them to share such – and *only* such – data with their State of incorporation. A multistakeholder approach on the model of the Bluetooth Special Interest Group which formed a quarter of century ago between core ICT companies such as Ericsson, Intel, Nokia, Toshiba, and IBM (Grillo and de Vries 2023) is therefore recommended, to ensure that the benefits of standardisation are shared as widely as possible among impending and potential industry actors, which would in turn foster States’ ability to apportion tax entitlements from global profits successfully. Major regulators such as the European Commission (2022, 111;120) as well as the Organisation for Economic Cooperation and Development (OECD) and other norm-setting organisations (WU Global Tax Policy Center 2022, 21) seem to be working towards such solutions already, not least by hosting debates among an unprecedent variety of public and private stakeholders. One more relatively recent phenomenon that this to-be-developed standard should cater for is that of cryptocurrencies; this would, however, take me too far, and is best suited for a self-standing paper.

I was writing *supra* that in the case of stateless individuals, providing them with travel documents that attest to their identity while not officially granting them or confirming their citizenship is going to help only to a very limited extent, in that several governments can be expected to not take such documents seriously enough – especially whenever the need of cross-border cooperation about such individuals arises. When it comes to *digital* nomads, the situation is reversed, and higher visa-free informality is often called for, with not only citizenship, but even identity information being depicted as unnecessary (Khanna 2021):

The origin of the passport is much less as a restrictive symbol of exclusive identity than a simple *laissez passer*: a request for safe passage. So how can we return to a world where passports don’t stand for who you are, but simply identify you on your way to where you’re going? The passports of the future should be based on skills and health rather than nationality. […] This early phase of the post-pandemic world provides a unique opportunity to *pre*-design the next migration system, one better suited to a global population constantly searching for political, social, and/or ecological stability. But to get there we’ll have to embrace blockchain technologies, and biometrics – which together would enable a decentralized global platform for individual mobility.

While skills and health are not wide enough criteria to encompass for the design of tomorrow’s future (“need” being another important variable, as extensively demonstrated *supra*), the cited passage does insightfully project us onto the new era of migration management, where borders lose significance while talent, necessity, and opportunity drive the crafting of a decentralised system of identity onboarding for all. Decentralised, however, is not yet tantamount to document-free. In this regard, it is in all States’ interests to negotiate standards that can speed up border procedures and identity processing. Destination countries will need to quickly verify an unprecedented amount and variety of foreign IDs against “templates”, and whenever a new identity onboarding was deemed necessary, States attracting and welcoming digital nomads will need to perform it into a format that can approximate most templates of relevance for the onboardee’s business lifestyle and travel habits, so as to reduce the burden for fellow “remoteness-friendly” jurisdictions. In these contexts, what authorities will need to ensure is that onboarding is not pursued or “hoped-for” by wealthy individuals to escape regulatory reach from other jurisdictions, in particular as related to so-called “white-collar” crimes, high-profile transnational mafia affairs, and money laundering.

## 8 Policy Recommendations

### 8.1 What Would Technical Standards Need to Ensure?

For an identity system to be run digitally, identification, authentication, and authorisation should all be performed digitally (Nyst et al. 2016, 28). Standards in this domain should be devised in such a way that government officials can access identity data but not alter it, nor should they be able to store it into or share and transfer it across multiple unrelated departments, including via simple measures such as restraints on taking screenshots. The ultimate “switch-off control” for each onboardee’s profile should rest with them, in such a way that if the regime changes or any other political upheaval places them at risk, migrants are allowed to straighforwardly delete their data as a last-resort option. If they do choose this extreme option, migrants should be under an obligation to create a new identity with which the new government can identify them; the whole point of this operation is to spare their *previous* records for being used by the new government for persecutory purposes stemming e.g. from tribal-ethnic rivalries and community disputes owing to the migrant’s affiliations with the *previously dominant* clan and ruling class.

On a more operational take, onboarding *devices* should be waterproof and heat-resistant as a minimum, and onboarding mobile *stations* should possibly be fireproof as well. Translation options shall be available from/to the six official UN languages and those most widely spoken by the regional staff of international organisations as well as local cross-border authorities – also accounting for and drawing on relevant legal provisions in cognate fields such as disaster recovery or diplomatic protocol. Extremely sophisticated recognition of synthetic faces and the like should be ensured: once confined to the realm of science fiction, these wearable silicon skin appliances are a widespread reality nowadays, and especially popular among smugglers, war criminals, and undercover governmental agents. Indeed, it is widely documented that large migratory flows are exploited by top criminals to clear their records and transplant their criminal undertakings elsewhere, as well as by secret services to access jurisdictions off-radar.

In designing these technologies, programmers should avail themselves of all the most accredited standards already being developed for remote digital know-your-customer (e-KYC) solutions, including cybersecurity and proof-of-authenticity protocols (Arab Monetary Fund 2019, PwC 2016, The Paypers 2020; Pic, Mahfoudi, and Trabelsi 2020). Additionally, they should consider making them compatible with satellite-delivered Internet, which is slightly slower and more expensive compared to cabled Internet, and subject to potential weather-dependent temporary disruptions, but it provides wider coverage, and – of special importance here – it stands less subject to political whims (cabled Internet and cell networks can be and indeed are switched off more easily by authorities – Feldstein 2022; Guest 2022; Vecellio Segate 2024, 336) in situations of spontaneous unrest and protest, which are so inherent to migratory management and especially characteristic of detention facilities for “unlawful” migrants. In the case of asylum seekers at high risk of retaliation, in particular, Internet networks powering onboarding stations should be as proprietary as possible, refraining from relying e.g. on public unprotected Wi-Fi hotspots for the transmission of sensitive identity data. Nevertheless, the political limitation is that States would expectedly not allow the release and even less the official adoption of such privacy-upholding technologies, which would need to be informally placed onto mobile carriers by NGOs and transported wherever needed while defying security checkpoints.

Another aspect to take care of regards the technical “updatability” of onboarding solutions in order to stay compliant with higher requirements or more streamlined processes as they arise (World Bank Group 2017, 27).15This means more generally that in fast-paced regulatory fields, technology solutions are to be provided under an understanding of the relevant policy trends, in order to accommodate probable forthcoming enhanced regulatory requirements without the need to drastically reset the solutions themselves. Narrow or legalistic compliance should be thus discarded, to favour “optimal” or “teleological” compliance whenever possible, and anticipate this way more demanding requirements as they may plausibly arise in the close future. In this sense, a State’s “advantage of backwardness”16On this concept in contexts of public-private technology deployment and rollout, see (Vu and Asongu 2020). might be key not only to first-time implement machines with the newest standards, but to test systems that could be comfortably made compatible with potential new standards immediately after. Updates should also be universally easy to access by end-users themselves, without linked mobile phones and other accounts that might change over time and place or expire; also, the availability of SIM cards, in the absence of ID documents, cannot be taken for granted, as demonstrated for instance by discriminatory policies against the Rohingya (Martin and Taylor 2021, 59). The token should be independent from any other device or service, and where it serves as the only, exclusive way to access services, it should provide for immediate assistance in the event of technical issues, such assistance (at least upon initial automated steps) being offered by readily available humans as opposed to robotic chatbots – not even anthropomorphic AI systems are going to be trusted (Zhang et al. 2023), or *should* be trusted (Vecellio Segate and Daly 2023, 11), especially where “functional literacy” and scholarly levels are sub-average. The linking of several public and private services into one single app or platform is deemed problematic by e.g. Aadhaar users (Krishna 2021; Thaker 2018), but immediate reaction by humans and the unlinking of the token from third devices should ease some of these concerns. Residual worries will be deep-seated in privacy violations, and for good reasons (Vecellio Segate 2022c, 335–336).

Whatever the technology solution, technical standards should define what information to capture, hide, transfer, display, and store in each circumstance, depending on the authorities’ trustworthiness and role, as well as on the applicable “soft” and “hard” laws – some of which have been discussed in this paper. There is no need to conceive for identities that are either fully displayed or fully concealed; far more efficient (and legally compliant) would be to devise concrete situations where certain pieces of information are displayed, captured, stored, and transferred, whereby others are ignored or kept hidden. What I am suggesting is to build on already existing situational frameworks17Refer for instance to the “Sarah at the nightclub” example within (UK Department for Science, Innovation & Technology 2023). and “specify” them for catering to the “borderline” situations I have being illustrating in the present work. This is also in keeping with the data-minimisation principle that is increasingly listed within major data-protection frameworks and compilations of best practices (Maple, Epiphaniou, and Bottarelli 2021b, 9; Vecellio Segate 2022b, 91), and that *a fortiori* applies in precarious (and regulatorily demanding) contexts of cross-border migration, disaster recovery, community resilience, development cooperation, crisis response, and humanitarian aid – among others (Gazi 2020, 3; Qadir et al. 2016, 16–17). *Functional* (purpose-specific, assumingly tokenized) and *foundational* identity onboarding (Hicks, Mavroudis, and Crowcroft 2022, 3), however, may require different thresholds of “minimum core” data to be shared with authorities, first responders, and other relevant parties even in these peculiar contexts. To exemplify, consider the attribute “age”: foundational ID may require its full disclosure, therefore all options will revolve around who should then access that information; instead, functional ID might be satisfied with either partial disclosure (say, the birth year alone), or no disclosure of this particular attribute at all, or a threshold-based confirmation that one’s age matches preset categories (e.g. older than *X*, or younger than *Y*).

As my last point here, I suggest that even those who do not wish their biometrics be recorded, should be provided with an identifying code as for at least one of their extended-family devices to be included within the national early-warning, emergency-alert programs – if applicable; needless to say, this turns out crucial in the context of humanitarian operations.

### 8.2 How can the Introducer’s Trustworthiness be Enhanced?

As obvious as it may sound, the first element to ensure that introducers best attend to situations of borderline citizenship is to provide for specific guidelines and regulations that delineate their role towards these situations, in accordance with all the considerations formulated above. Regrettably, this is not presently the case. If one considers, for instance, a quite detailed law that was recently enacted in the Philippines to regulate citizens’ biometric onboarding, paucity of information is supplied about “special circumstances”, with indigenous individuals and residents in remote areas being mentioned, but no reference whatsoever to the four categories scrutinised here. Even where the document accepts that ‘[a]n applicant who does not possess any of the documents […] shall be endorsed by a qualified Introducer’,18Section 8.C.4 of the Revised Implementing Rules and Regulations of Republic Act No. 11055 Otherwise Known as the “Philippine Identification System Act”, available at https://psa.gov.ph/sites/default/files/kmcd/Signed-Revised-IRR-RA11055-1.pdf; the same phrasing featured *verbatim* in the earlier version of the Rules, retrievable from https://psa.gov.ph/system/files/kmcd/IRR%20of%20the%20RA%2011055%20or%20PhilSys%20Law.pdf. no specific clause is inserted to regulate borderline situations vis-à-vis the introducer itself.

To start filling this gap, the reader is invited to consider the specific scenarios and complexities I have tried to single out above, for each category of individuals at “borderline citizenship” situations. All such scenarios, however, shall be problematised against evidence that ‘biopolitical technologies, such as biometrics, highlight, and heighten, the tension between care and surveillance’ (Iazzolino 2021, 112), which is of special relevance precisely to asylum seekers, stateless individuals, nomadic populations, and internally displaced people. The way we should account for mentioned tension is to make sure that when the introducer’s function is performed through biometric technologies, the latter are as user-centred and intrusion-protected as feasible. Otherwise, mistrust for introducers themselves (that is, for their role and profile) might couple with mistrust for the technologies they are equipped with to remotely onboard individuals.

### 8.3 In what Ways Should the Private Sector’s Overreach onto Standards’ Selection and Socialisation be Problematised?

The OECD, the G20, and other organisations and policy fora have long been advocating for a “smart” and “interconnected” data sharing across governmental departments, in order to reduce costs, optimise efficiency, and enhance data-analytics outputs (OECD 2015, 18–70; Wang 2018). One not-so-tacit implication is that whole-of-government approaches *within* governments will also foster data sharing *across* governments, i.e. internationally (e.g. Vecellio Segate 2022c, 201–351).

The benefits of “whole-of-government” approaches to data handling are often touted as self-evident and legitimate, not least in preventing data breaches (Carlin 2016). Developmental studies, too (e.g. Effah and Owusu-Oware 2021), lament the inefficiency inherent to data duplications in biometric systems between sector and national layers of domestic data governance. Yet, critical studies have been evidencing how this beneficial effect should *not* necessarily be assumed as given (Calo and Citron 2021). To begin with, restraining and disapplying the whole-of-government management practice would serve its share towards preventing regulatory capture: one captured department might well co-opt private actors into benefitted from citizens’ data, but if the latter was departmentally siloed, the private capturer would need to negotiate access conditions with each single department – which would hopefully prove overly resource-draining and time-consuming.

Moreover, each piece of data is released by individuals under the tacit assumption or express provision that it will serve the only purposes for which its release was consented to: pursuing “whole-of-government” approaches, especially in borderline contexts, might well represent a breach of trust and originate waves of chilling effect across populations and individuals in similar conditions, who will refrain from sharing their data out of mistrust for the algorithmic readability of what sociologists of crime like Haggerty and Ericson (2003) call “data doubles” (Ruckenstein 2014). The latter are “collated” pieces of data which were supposed to be handled independently but that once read together, may provide original insights or predictive patterns about an individual’s choices and behavior; they often defeat the teleology or any confidentiality upon which data sharing with the government is customarily premised. When the whole-of-government approach within governmental departments is coupled with revolving doors between public and private appointments (and related databases and technology solutions), it is probably wise to raise some concerns in relation to data being actually processed exclusively for the purposes it was first collected, and shared or read by no other entity.

The World Economic Forum (WEF) believes that biometrics gathering for digital identity should be driven by FIs worldwide, along the entire chain of service delivery, authorisation, attribute exchange, authentication, attribute collection, and standard development. They propose so under the (unreferenced) assertion that FIs would be entrusted with the highest confidence by “users” with regards to asset protection and information confidentiality (World Economic 2016, 23; 80; 89). Anybody who is familiar with the global governance of information, as well as with PIL, however, knows way too well that this is not the case. FIs are primary subjects and objects of regulatory outreach by the most powerful States, starting with the US; under the tag of *inter alia* “anti-money laundering”, tax agencies and several other enforcement bodies tend to assert their power extrajurisdictionally to compel identity disclosure, due diligence, and/or data handover. While this is not an issue in the absolute, it becomes so in contexts of borderline citizenship, whose actors already belong to the most vulnerable and marginalised fractions of the most fragile societies.

To begin with, US corporate conglomerates have no business interest in deploying biometrics technology in the contexts described here, to the extent that if such technology’s development is wholly outsourced to them, risks are that these populations and individuals will fall outside their scope of action, or will be anyway designed with other targets in mind and thus not tailored to the specific needs being expounded here. Even if they were covered, however, they would not necessarily be so for the right reasons, but rather to demark even more pre-emptively the perimeter of those who can “access” (territories and services) from those who cannot. Individuals at the borders of citizenship are unlikely to have their financial records completely clear (or to have any at all), which might well be due to political persecution, group discrimination, fear of reprisal, intergenerational oppressions, and mistrust in institutionalised asset preservation. Second, and from a more legal governance perspective, FIs are increasingly being resorted to as an instrument of economic coercion and subjugation, not least in the form of political sanctions (Bogdanova 2022, 55; Franco 2015; Peksen 2017).

No doubt exists that some of these sanctions may be justified on ethical grounds (e.g. when implemented to exercise pressure on, say, Russian oligarchs against their factual involvement in Ukraine’s invasion), but when the jurisdictional and procedural boundaries of international law are overstepped and the most powerful States adopt unilateral coercive acts, the risk is that innocent (or merely “situationally guilty”) individuals will be targeted as well. This is especially likely in contexts of failed States and frequent regime change, where regime representatives may be targeted with sanctions for actions carried out while in power, while finding themselves on the “borderline” side immediately after – also from a generational, family, or “dynasty” standpoint.

Soft law, with all its limits never to be discounted, shall fit into the regulatory toolbox as well. To exemplify, the 2018 Asia-Pacific Economic Cooperation (APEC) Nonbinding Principles for Domestic Regulation of the Services Sector commit APEC parties to adhere to principles of transparency, independence, and “good administration” vis-à-vis *inter alia* the adoption of technical standards (Asian Development Bank 2022, 224).

One theoretically suitable policy forum for discussion and negotiation, whose outputs can be legally binding although it is currently traversing an identity crisis, is of course the World Trade Organisation (WTO). Not only do technical standards need to abide by the Technical Barriers to Trade (TBT) Agreement, but in terms of intellectual property (IP), for example, Article VI(4) of the General Agreement on Trade in Services (GATS) demands WTO members to negotiate domestic regulation disciplines on qualification, licensing, and indeed technical standards. The TBT Agreement is of express significance here as compliance thereto is reported within most standards (applicable to digital onboarding) themselves, including privacy and cybersecurity standards ISO/IEC 27001:2022 and ISO/IEC 29100:2011. As hinted at, the WTO system is currently the site of deeply rooted institutional struggles among value- and interest-divergent “geoeconomic blocks”, not least due to frictions between the US and China, but from a legal perspective it still remains one of the very few platforms where high-level technical and policy discussions around standards can take place and result in the adoption of internationally binding measures. This might read even more promising if one considers that migrations today are tightly interconnected with human rights concerns and environmental considerations that WTO panels and diplomats tend to increasingly consider towards their adjudicatory and policymaking activities. One drawback to consider is that WTO lawmaking, too, has regrettably incorporated the general resort by international institutions to unaccountable sanctioning (Neuwirth and Svetlicinii 2015); these sanctions are directed at States, not individuals, but the latter will in any case be the ultimate effect-bearers.

Whatever the forum for discussion, one shall caution against the overreach of the private sector into the identification of the most appropriate standards for remote digital onboarding in borderline contexts. Private companies are intimately connected with abusive border patrolling (Davitti 2019; Davitti 2020) and too often entrusted with the management of immigrants detention facilities (e.g. Radziwinowiczówna 2022; Yang 2022), which is exactly where some of the identity onboarding procedures might take place; resultantly, the risk of deploying identity procedures as detention management practices makes privatised violence and brutality even more real than it already is – especially so when procedures are digitised and outsourced to corporations on remote, turning accountable faces into ‘structural indifference’ (Bigo 2023, 231) and necropolitics (Davies, Isakjee, and Dhesi 2017, 1273–1274). In light of the controversial role of private contractors in migration management, those corporations which manage migration detention facilities should *not* participate in the definition of standards for identity onboarding in borderline contexts. Indeed, while it is not feasible to completely exclude corporations – especially multinational ones – from standard-setting and regulatory practices (Graz 2018), one should still aim to inject seeds of transparency, professional ethics, accountability, and social responsibility into these processes by ‘regulating private regulators’ (Verbruggen 2019). This could be attained, for instance, via stringent rules against sliding doors, conflicts of interests, secretive (unregistered) lobby, and regulatory capture, and by sidelining those corporations which already exhibit a record of abuses *throughout the entire financing and supply chain*, and which are contracted for border patrolling and migration management in the same regions, insisting on the very same (segments of) populations the technology is being designed for. Decoupling corporate rule-enforcement from corporate rule-making functions might also contribute to debunking vested interests and preventing abuses (Van Loo 2020).

From a more economic (and specifically antitrust) perspective, while the lack of standards may well lead to a series of anticompetitive behaviors, including vendor lock-in effects (World Bank Group 2017, 6), enforcing privately selected standards onto all market participants based on market power or regulatory capture is equally detrimental to a healthy competitive environment. Recourse to open-source standards is therefore recommended, especially for *and in the interest of* developing countries where it is expected that several updates to proprietary software would otherwise be necessary (ibid., 26).

Lastly, when it comes to standard-essential patents on the hardware (and software, where patentable) of remote-onboarding stations and devices, their global licensing in fair, reasonable, and nondiscriminatory (FRAND) terms should account for the multijurisdictional nature of the aforementioned phenomena and refrain as much as possible from non-comity behaviors such as anti-suit injunctions. Moreover, licensing terms for “licensed” humanitarian actors (and the supply chain related thereto) should be favourably set at preferential rates.

## 9 Conclusions

People are on the move today as they always have been, but with a caveat: for the first time in human history, the planet is entirely fragmented into bordered areas we use to call “States” *and whose borders are factually impenetrable* due to extensive surveillance, monitoring, patrolling, and “defence” technology being deployed – where these are missing, that is just because natural barriers (like oceans, uncrossable rivers, extensive deserts, polar areas, or high mountains) are supposed to fence most migrants off on their behalf. This hyper-securitised parcellation of the globe comes exactly at a time when surreptitious forms of apartheid are most sensitive and escalating fast, including unprecedented expressions of class and especially climate apartheid (Clark and Mitchell 2021; Hall and Weiss 2012; McCordick 2021; Rice, Long, and Levenda 2021).

Deeper and context-sensitive research is therefore warranted to identify those identity onboarding technology solutions that can accommodate these vulnerabilities while still catering for States’ unfortunate resolution to manage migration flows at their borders. More specifically, *the role of the introducer deserves far more policy and legal space than it has enjoyed until today: no guidance on its function, profile, and procedures is traceable* in PIL documents, but not even within policy reports or the most recent domestic laws on migration matters! This work, drawing on sources from the entire spectrum of the social sciences, but mainly drafted doctrinally and addressed to sociolegal scholars, intended to mark the first step in this direction, but more “frontier” and interdisciplinary research is needed to capture the sociolegal implications of deploying identity onboarding solutions vis-à-vis individuals in “borderline” situations. For instance, KYC solutions for remote customer onboarding in the banking sectors are frequently illustrated with technical flowcharts, even within official policy documents (e.g. PwC 2018, 53); the same effort should be made with scenarios of borderline citizenship, where the remote identity onboarding of individuals becomes extremely complex technically and operationally, but also legally, diplomacy-wise, and “geopolitically”.

Pledged research should account for the distinctions and overlaps between identity, nationality, and citizenship; it should review the role and effectiveness of the introducer in borderline contexts across different socio-legal cultures through comparative ethnographic lenses; and it should formulate proposals for public-private partnerships towards defining inclusionary, accountable, and balanced standards to facilitate cross-border identity management. It should do so by mapping the complex geometries of regulatory power between the regulated and the regulators, especially at the interfaces between domestic and international standard-setting exercises, as reflective of a tension between local and somewhat “global” (Esty 2006) administrative cultures that under pressure from technology pace and complexity, tend to outsource to private transnational actors increasingly large fractions of their prerogatives. Finally, said further research should accomplish all these objectives through digital ethnographic fieldwork (Goulden et al. 2017) in war-torn areas, situations of identity-sectarian conflict, and humanitarian emergency settings, in order to acquire trust, ask questions that matter, and be informed with inclusionary and field-specific insights that can speak for those societies and groups at higher risk of displacement, resource-plundering (including land-grabbing), persecution, and systemic exploitation. Because of the paucity of legal-policy guidance as well as its technical complexity, and in light of the increasingly violent reality millions of humans live in, this is further research that cannot wait any longer.
